# Gastrotomy Twenty-one Hours after Rupture of the Uterus; Removal of a Dead Child from the Abdominal Cavity, with a Successful Result

**Published:** 1854-11

**Authors:** John T. Gilman

**Affiliations:** Portland, Maine


*Gastrotomy twenty-one hours after Rupture of the Uterus; Removal of a Dead Child from the Abdominal Cavity, with a Successful Result.* By John T. Gilman, M. D., Portland, Maine.—Mrs. Hickey, an Irish woman, aged about thirty years, small stature, spare habit, and delicate constitution, was taken with labor pains, her third confinement, early in the morning of September 24, 1853. Her previous labors were severe and protracted, especially the second, when the child was taken with forceps, while the mother was suffering with puerperal convulsions.

Her physician, on the present occasion, informed me that he was called at 10 A. M.; labor pains were then frequent and regular; the os uteri sufficiently dilated to admit the end of his finger; the membranes entire; the head presenting. Her pains increased in strength and frequency through the day, although they accomplished but little; the os, at 9 P. M., being rigid, and dilated only to the size of a quarter of a dollar; the head remaining in the superior strait.

At 11 P. M., after a pain of great severity, the patient complained, suddenly and urgently, of great abdominal distress; and there was an entire cessation of uterine pains. He, believing labor was suspended, and that his services would not be required for the night, left the patient at 12. He was summoned again at an early hour in the morning, and found her free from uterine pain, but having the same indescribable abdominal distress. Considerable flowing had occurred, and she was somewhat exhausted. On examination per vaginam, the presenting part was found to have receded—the head could not be felt. He administered stimulating drinks and ergot in repeated doses, with the hope that they would excite uterine action. Failing to accomplish this, and conscious that something of a serious nature had occurred to his patient, he called Dr. Durgin, of this city, in consultation. After making a careful examination, Dr. D. suspected that the uterus was ruptured, and the child had escaped into the cavity of the abdomen.

I was called to the case late in the afternoon of Sunday, with Drs. Daveis, Thomas, and Le Prohon. We found a rent of the uterus, extending from the os upwards and backwards, and the organ itself firmly contracted. No part of the child could be felt. The abdomen was enormously distended, and so tender that she could not bear the slightest pressure upon it. She was in great distress, and entreated earnestly for relief.

The deplorable situation of the patient was faithfully represented to her and her friends, and the operation of gastrotomy proposed as affording the best, if not the only chance of recovery, which was readily assented to.

The patient was removed from her bedroom to a spacious adjoining chamber, the temperature of which had been raised to about 80°, and placed upon a mattress resting on two firm tables. Pure sulphuric ether was first administered, but failing to produce its anesthetic effect, chloroform was substituted, and the patient soon brought under its influence.

The water having been drawn from the bladder by the introduction of a very small gum-elastic catheter, assisted by the gentlemen before mentioned, I made an incision through the abdominal parietes, commencing an inch above the umbilicus, and extending down along the inner edge of the left rectus muscle to within an inch of the pubes. The back of the child presented to the parietes, the head resting on the pubes.

The abstraction of the child and placenta was soon accomplished; coagula, and the fluids effused into the cavity of the abdomen, were expelled through the aperture. The divided surfaces were carefully brought together and secured by ligatures and adhesive straps, and a wide flannel swathe placed around the abdomen. The child was of large size and well formed, and had remained doubtless in the abdominal cavity from 11 P. M. Saturday, till 8 P. M. Sunday—a period of twenty-one hours.

The patient soon recovered from the effect of the chloroform, and expressed herself as being entirely free from distress; and not feeling so much exhaustion as after her previous confinements; and, indeed, the vital powers did not seem so much depressed after as before the operation. An opiate was administered, and our patient, hopeful and happy, was left in the care of faithful attendants for the night.

Monday morning, 26*th*. Patient slept well; free from pain; but little tenderness or fulness of the bowels; pulse 90, with good expression of countenance; evening, the same.

Tuesday morning, 27*th*. Pulse, 95; slept part of the night; abdomen more swollen and tender; directed an enema of castor-oil and the oil of turpentine, and the application of strong mercurial ointment to the abdomen, covered with oiled silk.

Evening. Enema operated well, and gave sensible relief.

Wednesday morning, 28*th*. Passed a sleepless night; increased abdominal swelling and tenderness; pulse 105; increased thirst; regurgitation of drinks from the stomach. Evening. Thirst and fever augmented by bronchial irritation and cough. Directed an ounce of castor-oil to be given with a drachm of oil of turpentine. Evening. Cathartic operated powerfully, and with great relief to the patient; pulse 105; bowels softer, and less tender; all the symptoms better.

Friday morning, 30*th*. Had a good night, with some quiet sleep; pulse 105. Iu the afternoon, all the distressing symptoms before enumerated returned with increased severity, and continued till 10 P. M., when a spontaneous diarrhœa came on, with very decided relief to the patient.

Saturday morning, *Oct.* 1. Pulse 100; diarrhœa continues; symptoms more favorable; patient pronounces herself better.

Sunday morning, 2*d*. Patient better; diarrhœa continues, but not to such a degree as to require any interference.

Monday morning, 3*d*. A decided improvement in every respect; all the symptoms highly favorable.

The patient continued to improve daily. The mercurial ointment was discontinued on the tenth day, having made a decided impression upon the system. The external wound, at that time, had united at several points, and presented a healthy appearance. The convalescence was rapid and uninterrupted. The patient was able to sit up on the fourth week, to walk about her chamber on the fifth, and to resume her domestic duties on the seventh.

At the present time, more than four months since the operation, Mrs. Hickey is in excellent health, and fully competent to discharge all the duties—laborious as some of them are—which belong to her humble condition in life.

In conclusion, I would briefly state that, in the treatment of this case, the mercurial impression was chiefly relied upon in its controlling effect upon inflammatory action. Opiates, in some form, were daily administered, and repeated as often as the comfort of the patient required them. The diarrhœa, which I attributed, in a great degree, to the action of the mercurial ointment, superseded the use of cathartics. Perfect quiet and rest were enjoined, and strictly observed. The diet, for the first two weeks, consisted of mucilaginous drinks, and occasionally of weak table tea. She afterwards partook freely and with great relish of grapes, and, on the twentieth day, of weak animal broths.

To my friends, Drs. Daveis and Thomas, I am greatly indebted for very valuable assistance rendered at the time of the operation, and during the subsequent treatment.—*Am. Journ. Med. Science.*

